# Comparative analysis of optimized logistic regression with state-of-the-art models for complex gastroenterological image analysis

**DOI:** 10.3389/fmed.2025.1655612

**Published:** 2025-11-19

**Authors:** Daniela-Maria Cristea, Ioan Sima, Laszlo Barna Iantovics

**Affiliations:** 1 University ‘1 Decembrie 1918' of Alba Iulia, Alba Iulia, Romania; 2 Doctoral School of Letters, Humanities and Applied Sciences, George Emil Palade University of Medicine, Pharmacy, Sciences and Technology of Targu Mures, Târgu Mureş, Romania; 3 Doctoral School in Mathematics and Computer Science, Babes-Bolyai University, Cluj-Napoca, Romania; 4 Electrical Engineering and Information Technology Department, George Emil Palade University of Medicine, Pharmacy, Sciences and Technology of Targu Mures, Târgu Mureş, Romania

**Keywords:** gastrointestinal polyps, colorectal disease, machine learning, logistic regression algorithm, multinomial classifier, random forest, k-nearest neighbors, support vector machine

## Abstract

**Introduction:**

Classifying gastrointestinal (GI) polyps detected in colonoscopy images is a critical task in colorectal cancer prevention. Given the diagnostic ambiguity of serrated polyps, which share morphological features with both hyperplastic and adenomatous lesions, this study focuses on multiclass classification using machine learning (ML) techniques. Multiclass Logistic Regression (LR), a model favored by clinicians for its interpretability, was initially optimized and evaluated.

**Methods:**

A structured dataset comprising 152 instances and 698 extracted features was used. We conducted a statistical analysis of 88 LR configurations, varying solvers, penalties, and regularization strengths. To improve classification performance, four additional ML algorithms were implemented: k-Nearest Neighbors (kNN), Support Vector Machine (SVM), Random Forest (RF), and XGBoost. For each classifier, parameter tuning was applied using grid search and stratified cross-validation.

**Results:**

The best-performing LR model (liblinear solver, L1 penalty, *C* = 0.01) achieved an accuracy of 70.39%, outperforming physician benchmarks (experts: 65.00%, beginners: 58.42%). In the multiclass setting, XGBoost achieved the highest macro-average F1-score (0.88) and overall accuracy (89.34%), followed by Random Forest (F1 = 0.85, accuracy = 86.05%), SVM (F1 = 0.83, accuracy = 84.21%), and kNN (F1 = 0.56, accuracy = 66.38%).

**Discussion:**

While LR remains valuable for its interpretability, ensemble methods such as XGBoost and Random Forest demonstrated superior performance and robustness. These findings support the integration of advanced ML models into clinical decision support systems, particularly in low-data scenarios where deep learning may be impractical.

## Graphical Abstract

**Figure:**
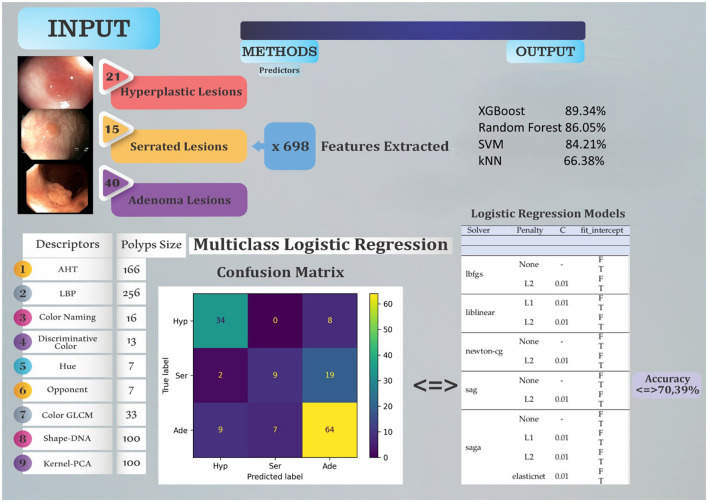
Overview of the machine learning pipeline for colorectal polyp classification.

## 1 Introduction

A colonoscopy involves a series of steps, starting with bowel preparation, wherein patients consume a specific solution to cleanse the bowel, ensuring optimal conditions for the procedure. Subsequently, a colonoscope equipped with light and a camera is inserted into the rectum for examination (1–4).

Regardless of colonoscopy's efficacy as the main screening technique for colon cancer, its limitations are apparent due to a high percentage of atypical malignancies and undiagnosed precancerous lesions, which suggests that more research is necessary (5, 6). Adenocarcinoma is the most common subtype of colorectal cancer, making up over 90% of all cases. Colorectal cancer can spread to both colon and rectal cancer (7).

Colorectal polyp detection models typically aim for one of three objectives: polyp segmentation (8, 9), detection (10, 11), or classification (12). Some models are trained to fulfill multiple objectives concurrently, such as detecting or segmenting polyps followed by classification (13). Detection models learn to localize polyps in images by processing training images alongside their labels, which typically include bounding box coordinates and polyp presence indicators. Segmentation models, conversely, are trained to outline polyps in colonoscopy images using the corresponding image masks as labels (14). Lastly, classification methods identify polyps without determining their location.

Feature extraction is a fundamental step of image analysis, especially in the field of medical imaging (15), where accurate diagnosis and treatment depend to a large extent on the interpretation of visual data. Gastroenterological studies, in particular, necessitate complex feature extraction techniques due to the complex nature of gastrointestinal pathologies (16). This study examines feature extraction techniques designed specifically for the analysis of gastroenterological images, including the examination of 2D texture, 2D color, and 3D shape features.

Classification of complex lesions in real time (6, 17) presents a challenge due to the slight distinctions between benign and malignant lesions. Colonoscopies currently utilize two main imaging techniques: Narrow Band Imaging (NBI) and conventional White-Light (WL) high-resolution endoscopy (18, 19). NBI, employing green and blue wavelengths, enhances visualization by highlighting blood vessels, aiding in lesion characterization. WL endoscopy evaluates polyps on the basis of their appearance under white light, sequentially predicting histology.

During colonoscopies, physicians capture images of tumor masses known as polyps, which necessitate accurate classification as either benign or malignant to guide appropriate treatment decisions. Various classification systems with some limitations such as Kudo (20), Sano (21), and Paris (22) are employed for this purpose. However, the reproducibility of data among endoscopies remains a challenge, particularly with the Paris classification system.

Tian et al. (11) explore the use of a Deep Neural Network for detecting, localizing, and classifying polyps in videos obtained during colonoscopies. The proposed model utilizes a flexible endoscope to capture video images of the colon, which are then automatically analyzed to identify potential anomalies, such as polyps, which are precursors to colorectal cancer. It is based on Convolutional Neural Network (CNN) architecture and includes a two-stage method for detecting and classifying polyps, using the Softmax function to determine whether a region in the image belongs to a polyp or non-polyp category. The model's performance is evaluated using metrics like ROC AUC, which helps assess its ability to distinguish between classes.

Qinwen et al. (23) focus on the creation and assessment of various ML models to predict colorectal polyps based on electronic health records. It concludes that ML models, particularly AdaBoost, offer a non-invasive and cost-effective strategy for predicting colorectal polyps, guiding screening and treatment decisions.

Kim et al. (1) uses a range of annotated colonoscopy datasets to train AI models, focusing on the clinical relevance of data from actual colon and rectal surgical procedures. Image features include color, texture, and edge-based characteristics for distinguishing polyps from other GI structures.

Tasdemir et al. (24) use both a custom dataset and the UniToPatho dataset, which include histopathological images labeled by expert pathologists. Key features extracted include contrastive representations that enhance the model's ability to distinguish between adenomatous, tubular, and tubulovillous polyps. The authors employ Supervised Contrastive Learning combined with the Big Transfer (BiT) model, which leverages pretrained CNNs to improve classification, especially in cases with limited labeled data. Achieving classification accuracies of 87.1 and 70.3% on the custom and UniToPatho datasets, respectively, the model outperforms traditional CNNs by leveraging in-class and out-of-class image distinctions, providing more reliable polyp subtype classification.

Hmoud et al. (25) utilizing the Kvasir dataset, which includes 5,000 images across five GI disease categories, including polyps; provides a robust dataset for lower GI disease classification. The study focuses on deep color, texture, and shape features that are essential for accurate classification within the CNN architecture. The research applies pretrained CNN models, including GoogleNet, ResNet-50, and AlexNet, with transfer learning to adapt the models to polyp detection. Image augmentation is used to enhance the training process. The pretrained CNNs achieve high accuracy, with AlexNet reaching 97%, demonstrating the effectiveness of CNNs in GI disease classification and underscoring their potential to match or exceed physician diagnostic performance.

In Cincar and Sima (26), four ML algorithms were applied: Support Vector Machine (SVM), Random Forest (RF), Random Subspaces (RS) and Extra Trees (ET), of which RF performed best.

Model fit is an important subject that must be studied in rigorous research based on ML, which is the case of mLR hyperparameter tuning, a difficult task. In this research, we studied the state-of-the-art LR algorithm most frequently applyed by medical researchers to determine the best-fitted hyperparameter tuning for the problem of GI polyp image classification in adenomas, serrated and hyperplastic which is difficult even for physicians. It must be noticed that serrated are difficult to define, since they reveal features of both hyperplastic and adenoma; based on this fact, they are difficult to identify accurately even by expert physicians (27). Serrated lesions can cause 8%–15% of all colorectal malignancies. We chose to experiment on a well-known dataset, with easy access through a web interface, taken from Mesejo et al. (12).

This research used a benchmark dataset of colorectal polyps comprising 152 instances, corresponding to 76 polyps annotated with three lesion types: *hyperplastic, serrated*, and *adenoma*. A total of 698 individually made features were extracted from image-level data, including morphological, textural, and intensity-based descriptors. These features served as input for a series of ML classifiers, rather than applying models directly to raw images. Both multiclass and binary methods were used to address the classification task. In addition to Multiclass Logistic Regression (mLR), four state-of-the-art algorithms were implemented and evaluated: Random Forest, k-Nearest Neighbors (kNN), Support Vector Machine (SVM), and eXtreme Gradient Boosting (XGBoost). The comparative analysis focused on macro-average F1-score, lesion-specific metrics as benchmarks.

The contributions are the follows:

1. Conducted an extensive evaluation of mLR, including the optimisation of 88 parameter configurations varying solvers, penalties, regularization and a comparative analysis against physician expert and beginner performance on colorectal polyp classification.
2. Proposed a decision rule to guide model selection based on lesion-specific performance, enabling transparent comparison between mLR and other state-of-the-art classifiers.
3. Implemented and benchmarked five ML algorithms, mLR, kNN, SVM, Random Forest, and XGBoost, for the classification of colorectal polyps into three lesion types: *hyperplastic, serrated*, and *adenoma*, using a reference dataset of 152 instances and 698 extracted features.
4. Our comparative analysis demonstrated that ensemble methods, particularly XGBoost (macro-average F1-score = 0.88, accuracy = 89%), outperform both traditional classifiers and previously published models such as the Random Forest approach of Mesejo et al. (12, 26).
5. We provide a reproducible evaluation framework that integrates multiclass and binary classification metrics, enabling fair comparison across models and lesion types, and supporting future benchmarking efforts in medical image-based polyp classification.

The subsequent sections of the paper are structured as follows. Section 2 presents the dataset, multiclass logistic regression and performance metrics used in this work, conducted in the GI polyps classification from colonoscopy images and the proposed methodology employed in this study. In Section 3, the results of experimental investigations are presented, comparing the methods of physicians with levels of experience. Sections 4 and 5 presents the discussions, conclusions and future work.

## 2 Materials and methods

### 2.1 Colonoscopy dataset

The trustful, accurate colonoscopy dataset is a complex video and picture dataset of 76 polyps, that was introduced in Mesejo et al. (12). The dataset consists of 152 brief colonoscopy videos and 152 photos (768 × 576 pixels) and 698 features extracted from every photo.

**Figure 1:**
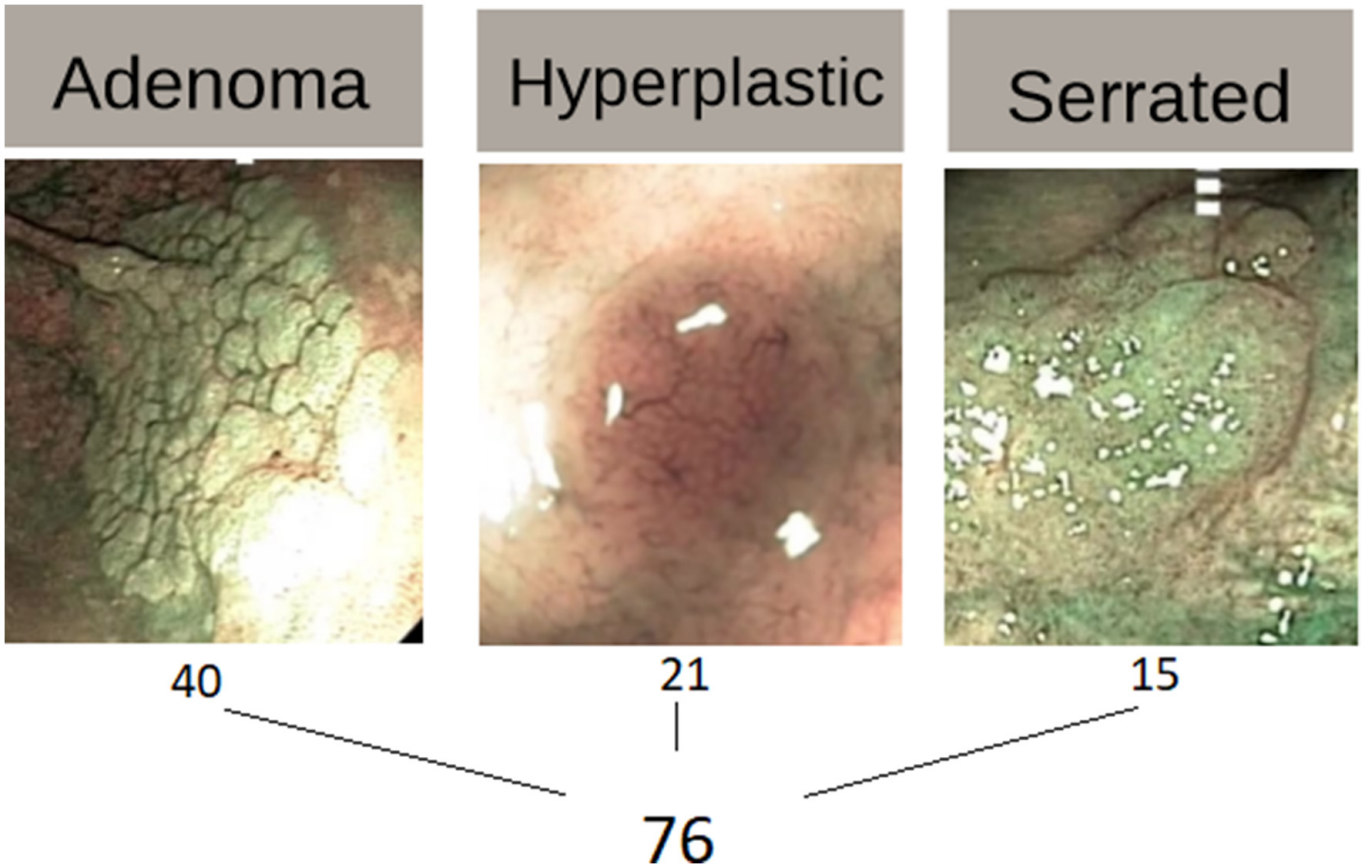
Example of gastrointestinal (GI) polyps types.

The polyps in this dataset are divided into three categories: 21 hyperplastic, 40 adenoma, and 15 serrated lesions. As part of their everyday protocol, the physicians captured two pictures during colonoscopies using WL and NBI, respectively, for every polyp, they obtained one WL image and one NBI image, resulting in 42 images for hyperplastic, 80 images for adenoma, and 30 for serrated ones. Mesejo et al. (12) extracted 698 features from each image as follows: Amplitude Histogram of Texture (AHT)—166 features; Local Binary Patterns (LBP)—256 features; Color Naming—16 features; Discriminative Color—13 features; Hue—7 features; Opponent—7 features; Color Gray-Level Co-occurrence Matrix (GLCM)—33 features; Surface Signatures with Shape-DNA—100 features; 3D Cloud Signatures with Kernel-Principal Component Analysis (PCA)—100 features. The beginner and expert opinions are only based on endoscopic image data without knowing any other clinical information, meaning that the medical physicians were tested similarly with the same data as the ML algorithms. Figure 1 illustrate example images for each polyp type, providing a visual understanding of their unique structural characteristics.

In Figure 1, the three columns illustrate example images for each polyp type, providing a visual understanding of their unique structural characteristics.

- Adenoma polyps type, shown in the leftmost image, represents polyps with a higher likelihood of developing into cancer, making them crucial targets in colonoscopy screenings. This study included 40 adenoma instances, highlighting the prominence of this polyp type in the dataset.
- Hyperplastic polyps. The image shows hyperplastic polyps, which are generally considered benign and have a lower risk of malignancy. There are 21 instances of this type in the dataset, providing sufficient samples for evaluating the model's ability to distinguish it from the other types.
- Serrated polyps shown in the rightmost image, serrated polyps are unique because they have a mix of features seen in both adenoma and hyperplastic polyps, making them particularly challenging to classify. With 15 instances in the dataset, serrated polyps are known to be more difficult for both ML models and physicians to identify accurately due to their ambiguous characteristics.

Each type is labeled with its corresponding sample size, totaling 76 polyps across the three categories. This figure was included to clarify the diversity and complexity of the polyp types being analyzed in the study.

### 2.2 Feature extraction

For every image, a rich dataset of 698 features, including 2D texture, 2D color, and 3D shape descriptors, are obtained, providing a gastroenterological image characterization.

#### 2.2.1 2D texture features

Gastroenterological images frequently have complicated textural differences that provide key diagnostic information. Invariant Gabor Texture Descriptors (AHT) (28) and Invariant Local Binary Patterns (ILBP) (13) can capture accurate texture differences. AHT, based on Gabor filters, improves in storing texture information, uncompromised by illumination, rotation, or scaling changes. In contrast, ILBP gives binary representations of texture patterns, allowing texture analysis in a shorter time.

#### 2.2.2 2D color features

Color information in gastroenterological images can provide a helpful understanding of tissue features and anomalies. Hue and Opponent histograms (29), coupled with Color Gray-Level Co-occurrence Matrix (Color GLCM), Discriminative Color (30) and Color Naming (31), create a full collection of color characteristics. These descriptors accurately capture color variations, semantic color distributions, and discriminative color features, allowing for an exact definition of GI lesions and tissues.

#### 2.2.3 3D shape features

The three-dimensional shape of GI polyps has important diagnostic implications. 3D reconstruction utilizing Structure from Motion (SfM) (32) approaches enables faster retrieval from data. Shape DNA (33) and Kernel PCA (34) are effective techniques for representing polyp surface signatures and spatial architecture. These descriptors allow the characterization of non-rigid structures while also making use of the confusing spatial connections found in gastroenterological images. The first row contains the video name and the second row is the ground truth, the real diagnosis of hyperplastic, Serrated and Adenoma, does not depend on the physicians assumptions based on images. The features extracted are the following (12):

- Amplitude Histogram of Texture (AHT)—166 features. This texture descriptor captures information about the distribution of amplitudes in images.
- Local binary patterns (LBP)—256 features. This texture descriptor measures the local distribution of binary patterns in an image.
- Color naming—16 features. These represent descriptions of colors in images, classified into a predefined set of color names.
- Discriminative color—13 features. This descriptor captures information about dominant and discriminative colors in images.
- Hue—7 features. These are characteristics describing the predominant color hues in the image.
- Opponent—7 features. This descriptor refers to the representation of colors in an opponent color space.
- Color gray-level co-occurrence matrix (GLCM)—33 features. This is a descriptor that analyzes the distribution of textures and contrasts in images.
- Surface signatures with shape-DNA—100 features. This descriptor refers to the representation of object shapes in images using a DNA-based model.
- 3D Cloud Signatures with Kernel-Principal Component Analysis (PCA)—100 features. This is a descriptor that performs a principal component analysis on a dataset in an implicitly defined feature space generated by a kernel.
- Within the output file, the first row contains the movie name associated with the image, and the second row contains the ground truth value associated with the image. This information can be useful in evaluating the performance of the models or algorithms used in the work.
- By using this output file, we have access to a detailed and multidimensional representation of the images, which can be used in various image analysis and recognition applications.

### 2.3 Methods related to LR all the aspects

LR is a supervised ML algorithm, used for binary or multiclass classification. mLR is a generalization of binary logistic regression that models the probability of each class using a softmax function. It is widely used in clinical research due to its interpretability and simplicity (35). The probability of a particular outcome is modeled using the logistic function, which takes the following form (36, 37):


$$\begin{array}{l} \begin{array}{c} p\left(X\right)=\frac{e^{\beta_{0}+\beta_{1}X}}{1+e^{\beta_{0}+\beta_{1}X}} \end{array} \end{array}$$ (1)

where *p*(*X*) denotes the probability that the dependent variable is equal to 1, given the value of the independent variable *X*. β_0_ is the intercept, representing the predicted log-odds of the outcome when *X* = 0. β_1_ quantifies the effect of the independent variable *X* on the log-odds of the outcome. *e* refers to the base of the natural logarithm, ensuring that the function's output is constrained between 0 and 1. Through some manipulation of Equation 1, we find:


$$\begin{array}{l} \begin{array}{c} \frac{p\left(X\right)}{1-p\left(X\right)}=e^{\beta_{0}+\beta_{1}X} \end{array} \end{array}$$ (2)

where $\frac{p\left(X\right)}{1-p\left(X\right)}$, known as the *odds*, represents the ratio of the probability of an event occurring to the probability of it not occurring. While probability *p*(*X*) ranges between 0 and 1, odds range between 0 and ∞. Odds close to 0 indicate a low probability, whereas odds approaching ∞ indicate a high probability. Odds and probability values are nearly the same for rare (unusual) occurrences when *P*(*X*) is very small, because 1−*P*(*X*)≈1.

The left side of Equation 2, or the log odds, is obtained by taking the logarithm of both sides. A linear logit is created through the LR model in X.


$$\begin{array}{l} \begin{array}{c} \log\left(\frac{p\left(X\right)}{1-p\left(X\right)}\right)=\beta_{0}+\beta_{1}X \end{array} \end{array}$$ (3)

Specifically, the output, ranging from 0 to 1, can be interpreted as the probability of belonging to class 1. This hypothesis delineates a soft boundary in the input space, assigning a probability of 0.5 to inputs at the boundary's center and tending toward 0 or 1 as we move away from it.

For LR, due to its non-linearity, there is no direct analytical solution for optimizing the loss function (log-likelihood). For its maximization, are used the methods: Limited-memory BroydenFletcher GoldfarbShanno (*lbfgs*), Library for Large Linear Classification (*liblinear*), Newton Conjugate Gradient (*newton-cg*), Newton's Method with Cholesky Decomposition (*newton-cholesky*), Stochastic Average Gradient (*sag*) and Stochastic Average Gradient with Acceleration (*saga*).

The *(lbfgs)* (38) solver is an analog of Newton's Method, and the *liblinear* (39) optimizes the loss function by iterating over each coefficient of the model and adjusting it while the other coefficients are held constant. The *newton-cg* is a solver that uses the conjugate gradient Newton-Raphson method for optimization, while *newton-cholesky* solver is a variant of the Newton-Raphson method that uses the Cholesky decomposition to solve the system of equations derived from the Newton-Raphson method. The *Sag* (40) and *saga* (a variant of *sag*) (41) are solvers that use stochastic variants of gradient optimization and they are efficient for large and sparse datasets. Unlike the other solvers, *saga* supports both L1 and L2 regularization as well as their mixture (ElasticNet).

### 2.4 Other methods compared with LR

kNN is a non-parametric algorithm that classifies instances based on the majority label among the *k* closest training samples. KNN is preferred for classification tasks requiring labeled data but can be sensitive to feature scaling and class imbalance. It has been applied in medical imaging tasks for its ease of implementation (42).

SVM constructs hyperplanes in a high-dimensional space to separate classes with maximum margin. The RBF kernel was used in this study to capture non-linear relationships. SVMs are known for their robustness in high-dimensional biomedical datasets (43).

Random Forest algorithm, proposed by Breiman (44), is an ensemble method that builds multiple decision trees and aggregates their predictions. It reduces overfitting and improves generalization, making it suitable for noisy medical data. In this study, RF was tuned using n_estimators=100 and max_depth=5.

XGBoost is a scalable and regularized boosting algorithm that builds additive models. It has shown superior performance in structured biomedical datasets and was the best-performing model in this study (45).

### 2.5 Performance metrics

To evaluate the quality of the multinomial classification model prediction, we used (1) Confusion Matrix and the metrics: (2) Accuracy, (3) Precision, (4) Recall (Sensitivity), (5) F1-Score, and (6) Macro-Average (26).

Confusion matrix is a tool used in the ML field to evaluate the performance of a classification algorithm. This is a square matrix of *n*×*n* size, where *n* represents distinct class numbers. Every element (*i, j*) of the array indicates the number of instances in the class *i* that were classified as belonging to the class *j*.

In the case of multinomial classification problems, the confusion matrix expands to include all classes of the problem. Table 1 exemplifies a confusion matrix for a problem with three classes.

**Table 1:** Confusion matrix for three-class classification.

**True/Predicted**	**Class A**	**Class B**	**Class C**
Class A	*T*_11_	*F*_12_	*F*_13_
Class B	*F*_21_	*T*_22_	*F*_23_
Class C	*F*_31_	*F*_32_	*T*_33_

where: (1) *T*_*ii*_ are True for class i (A, B, and C, respectively); (2) *F*_*ij*_ are False, that is class *i* is wrongly predicted as belonging to class *j*.

F1_Score—Harmonic mean of Precision and Recall. It is useful when there is an imbalance between the number of items of classes of the given problem, providing a unique measure of a model's performance by combining precision and recall.

Macro_average—arithmetic mean of Precisions, Recalls and F1-Scores, respectively. It is helpful in imbalanced datasets where some classes are under-represented because it gives equal weight to each class. This avoids situations where the model's performance on large classes dominates the overall evaluation and provides better clarity on the model's performance for all classes.

ROC AUC is a metric used in statistics and ML to evaluate the performance of a classification model. The Receiver Operating Characteristic (ROC) is a curve plotting the True Positive Rate (TPR) against the False Positive Rate (FPR) at various classification thresholds. TPR is just Recall (ratio of true positive (TP) over all positive (P)), presented above, and FPR is ratio false positive (FP) over all negative (N).

Area Under the Curve (AUC) measures the area under the curve, providing a scalar value that summarizes the model's performance. An ideal model has an AUC of 1, indicating a perfect separation between positive and negative classes in the binary case. An AUC of 0.5 implies performance equivalent to random guessing.

Unlike binary classification, where ROC AUC measures the model's ability to distinguish between two classes, specific methods are used for multiple classes: (a). One-vs-Rest (OvR)—the multi-class problem is broken into multiple binary problems, and for each class, the model evaluates how well it can separate that class from all others. Then, an average of the AUC scores for each class is computed; (b). One-vs-One (OvO) approach—classifiers are built between each pair of classes, and the AUC values are combined to obtain an overall metric. ROC AUC for multi-class classification is valuable for comparing models, especially when the class distribution is imbalanced.

### 2.6 Addressing the imbalanced dataset problem

Imbalanced datasets are common in ML fields. There are 76 polyps (see Figure 1) and, for every polyp, there are two photos, in NBI and White-Light. Thus, in the dataset exist 80 adenoma (Ade), 42 hyperplastic (Hyp) and 30 serrated (Ser) medical images. The issue is that using an imbalanced dataset for model building can lead to the wrong prediction (especially Accuracy) by favoring classes with more instances.

To appreciate the degree of imbalance in dataset, in multiclass classification problems, we define the *general imbalance ratio (IR)* and the *class imbalance ratio (IR(i))*:


$$\begin{array}{l} IR=\frac{max\left\{n\left(1\right),..,n\left(k\right)\right\}}{min\left\{n\left(1\right),..,n\left(k\right)\right\}} \end{array}$$ (4)

$$\begin{array}{l} IR\left(i\right)=\frac{n\left(i\right)}{min\left\{n\left(1\right),..,n\left(k\right)\right\}} \end{array}$$ (5)

where: *n(i)* represents the number of instances of class *i* and *k*—no of classes.

Thus, we obtained a general IR (IR = 2.66), an hyperplastic IR [IR(1) = 1.4] and an adenoma IR [IR(3) = 2.66]. Note that in this case the IR = IR(3).

Although there is no theoretical threshold concerning the exact degree of class imbalance, in practice, we consider that IR values lower than 2 are only marginally imbalanced and that dataset with an imbalance ratio of about 10:1 would be modestly imbalanced. The IR indicators show us a relatively small imbalance, which can be translated, anticipatively, by a small change in accuracy.

This problem of imbalanced data can be addressed in more ways: (1) Undersampling, (2) Oversampling, and (3) using the Class Weights. Since the dataset is small, we avoided using the undersampling method because it would have reduced it even more (from 152 to 90 images). As one of the initial design goals of this article was not to use synthetic data, the oversampling method is not suitable for addressing the imbalanced dataset problem. Thus, to approach this problem, the most appropriate method remained the method of using class weights. This variant is relatively easy to implement using the Scikit-learn library (46), because *class weights* is a hyperparameter of the LR algorithm (47).

Class weights are reversely proportional to the frequency of the respective classes. Each category would seem to have the same amount of weight if class weights were used. The class weights are generally calculated using the formula shown below:


$$\begin{array}{l} w\left(i\right)=\frac{n}{k\cdot n\left(i\right)} \end{array}$$ (6)

where: *i* —no of class; *w(i)*—class weight of class *i*; *k*—no of classes; *n*—total number of observation from dataset; *n(i)*—no of instances of class *i*.

Thus, we obtained: for Hyp: w(1) = 1.206, for Ser: w(2) = 1.688, and for Ade: w(3) = 0.633.

With the above calculated values of class weight, the experiments were performed for the balanced case.

### 2.7 Experimental design

#### 2.7.1 Proposed dataset preparation

Due to the smallness of the dataset, we chose not to use a separate test set, but only randomly extracted test sets at each run in the cross-validation stage. To avoid overfitting, we employed four-fold cross-validation. We chose the fold-fold cross-validation because the dataset is relatively small. This method involves dividing the dataset, consisting of 152 rows, into four equal folds, each containing 38 rows. Each model runs four times, with a different fold designated as the test set in each iteration, while the remaining three folds are used for training.

Because the original dataset was ordered according to the label, we first shuffled the rows randomly and for reproducibility, we used *random_state=42*. Random_state is a parameter used in Scikit-learn (46, 47), to control randomness in various procedures. It is a way to set a seed for the random number generator, thus ensuring reproducibility of the results. In the KFold context, random_state controls the shuffling of the data before it is split into folds for training and test, respectively. When re-running experiments, using a fixed random_state ensures that data splits and results will be consistent.

#### 2.7.2 Hyperparameters tuning

During each run, several performance metrics were calculated, including *accuracy, precision, recall, F1_score*, and *macro_average*. After completing all four runs, the average accuracy was computed to provide a robust estimate of the model's performance. Our experiments utilized both non-normalized and normalized data.

The parameters of the model are the coefficients of the following equation: *b*_0_+*b*_*i*_*X*_*i*_, where $i=\bar{1,n}$ and *n* = 698, with *n* being the number of features.

We chose to tune all hyperparameters Logistic Regression algorithm that the Scikit-learn library (46) provides, and which are relevant for this problem:

- *multi_class type*: Determines the strategy for handling multi-class classification.
- *solver*: Specifies the algorithm to use for optimization.
- *penalty*: Indicates the type of regularization applied to prevent overfitting.
- *C*: Controls the trade-off between achieving a low training error and a low testing error.
- *fit_intercept*: Decides whether to include an intercept term in the model.
- *max_iter*: Sets the maximum number of iterations for the optimization algorithm to converge.
- *class_weight*: Sets the LR algorithm used for balanced or imbalanced dataset.

To address the multiclass classification problem, we utilized both Multinomial (Softmax Regression) and OvR LR to extend the binary LR method (48) for handling multiclass scenarios with more than two discrete outcomes, employing five out of the six solvers available in Scikit-learn (46) LR: *lbfgs, liblinear, newton-cg, sag* and *saga*.

Regularizers were used to prevent model overfitting. The LR class from the Scikit-learn library allows two types of *penalty*: L1 and L2. Therefore, we have four values for the penalty hyperparameter: “L1," “L2," “none," and “elasticnet" (a combination of both L1 and L2). For models with the penalty value set to “elasticnet," the hyperparameter *l1_ratio* balances the L1 and L2 penalties. It must be a value between 0 and 1, where 0 corresponds to using only L2 regularization, and 1 corresponds to using only L1 regularization. We used a *l1_ratio* value of 0.5, which combines both L1 and L2.

The hyperparameter *C* is a positive value that represents the inverse of regularization strength, i.e., smaller values mean stronger regularization, *fit_intercept* specifies whether a bias (a constant value) is added to the logistic function, and *max_iter* represents the maximum number of iterations for the solver's convergence.

We used both the GridSearchCV library of Scikit-learn (46) for hyperparameter tuning and manual experiments. The adjusted hyperparameters are shown in Table 2. Theoretically, this results in 2 × 2 × 5 × 4 × 5 × 2 × 8 × 1 × 2 = 12, 800 experiments (the cardinal number of the Cartesian product of the nine sets). However, since not all combinations are compatible with each other, only 4,992 experiments remain.

**Table 2:** Hyperparameter tuning for all classifiers.

**Classifier**	**Hyperparameters considered**
Logistic regression	Data type {non-normalized, normalized}
	Multi_class type {multinomial, ovr}
	Solver {lbfgs, liblinear, newton-cg, sag, saga}
	Penalty {none, L1, L2, elasticnet}
	C {0.01, 0.1, 1, 10, 100}
	Fit_intercept {False, True}
	Max_iter {100, 150, 200, 250, 300, 1,000, 5,000, 10,000}
	l1_ratio {0.5}
	Class_weight {None, balanced}
Random Forest	{n_estimators: 100; max_depth: 5}
kNN	{n_neighbors: 5}
SVM (RBF kernel)	{gamma: 'scale'}
XGBoost	{n_estimators: 100; max_depth: 5; learning_rate: 0.1}

**Figure 2:**
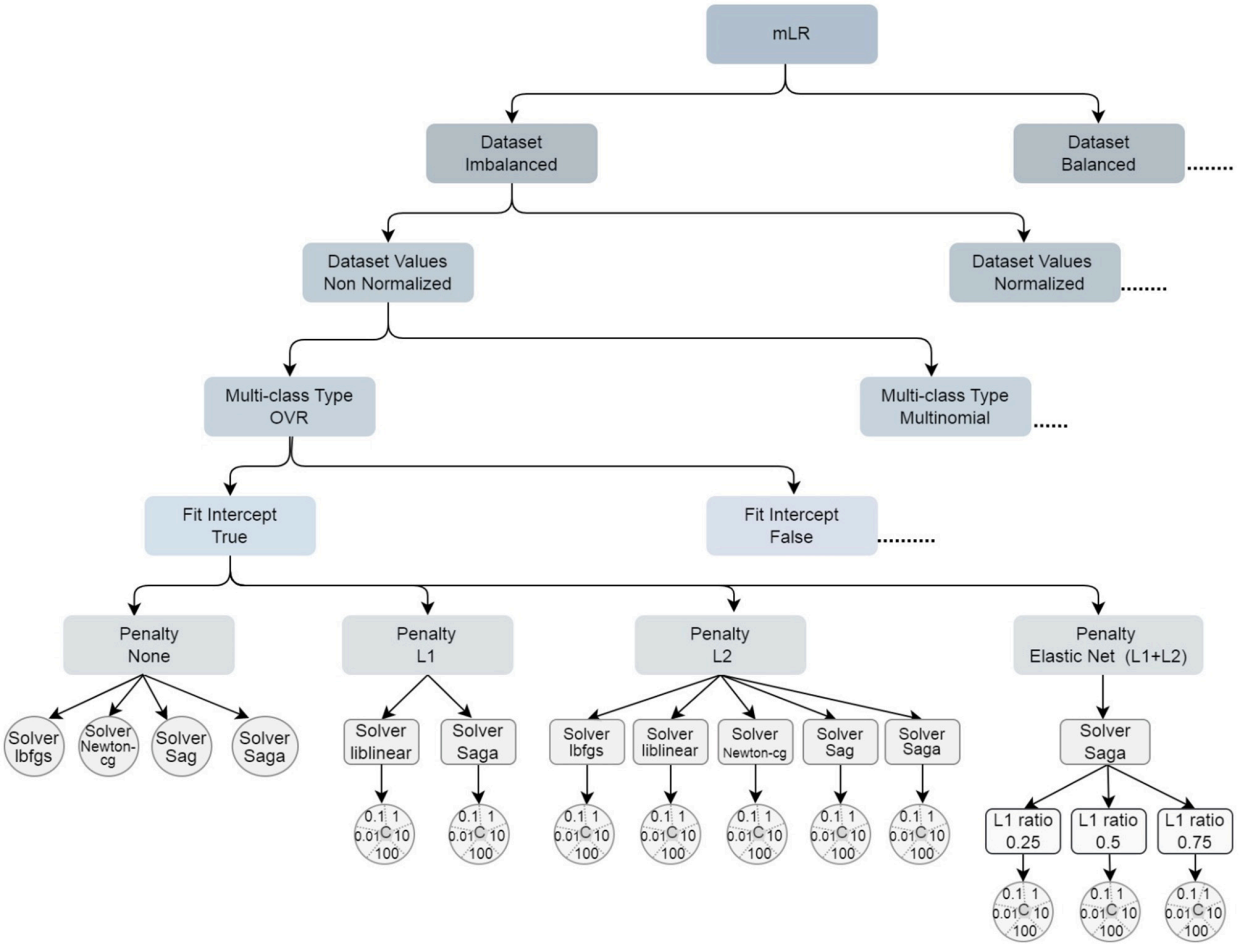
Decision rule for the hyperparameters tuning.

Figure 2 shows the decision rule for the hyperparameters tuning. The suspension points indicate that the sibling branch is symmetrical, based on this fact they are not included in the figure. The leaf nodes are represented by a circle, whereas the others are represented by rectangles with rounded corners. Since the hyperparameter *C* has 5 values to visualize them appropriately, they were included in a circle.

Sibling branches are symmetrical except for Multi-class type multinomial node, where the liblinear solver is not supported.

Since higher values of the hyperparameter *C* result in decreased accuracy, we have chosen to present only the models with *C* = 0.01 or the models that do not use regularization. As the algorithm required a large number of iterations to converge for most of the models, we have only included variants with *max_iter* set to 10,000.

It is known that when *n*_samples < *n*_features, *Dual* formulation is preferred to be set to True. During testing in dual formulation, the algorithm does not converge. Therefore, regarding this hyperparameter, we concluded that it cannot be used. The tolerance for stopping criteria (*tol* hyperparameter) was increased only if the algorithm did not converge. The following hyperparameters: *intercept_scaling, verbose* and *warm_start* did not influence the confusion matrices and therefore, none of the metrics. *n_jobs* is used only in parallel programming to set the number of CPU cores. In the case of our dataset, it was necessary to use. However, we recommend its use for larger datasets. Therefore, 176 models remain.

## 3 Results and discussion

### 3.1 Algorithm testing results

We conducted our experiments using Python 3.11.9, Scikit-learn library (46, 47), version 1.4.2, Matplotlib (49), version 3.7.5, NumPy (50) 1.26 version, Pandas (51), version 2.0.3.

In Table 3, the results of the 88 models tested on the imbalanced dataset are presented. The columns are defined as follows: (1) *Solver*—lists the solvers used for optimisation in the mLR models; (2) *Penalty*—specifies the types of regularization penalties applied to the models. There are four penalty types included, which affect how the model handles regularization; (3) *C*—we specified the value of the *C* hyperparameter (0.01), and in the case of models without regularization, this is meaningless, so it was not used, which is indicated by the symbol “–"; (4) *fit_intercept*—denotes whether the model includes an intercept term. There are two options: including an intercept (“T"—True) or not including an intercept (“F"—False); (5) *Accuracy (Std Dev)*—provides the accuracy and standard deviation of the models, expressed as a percentage. It reflects the performance and variability of the model's accuracy across four-fold cross-validation. This column is split into two other columns: “OvR" (Over-vs-Rest) and “Multinomial”. Each column, in turn, is divided into two columns, corresponding to normalized (“Norm”) and non-normalized (“non-Norm") data, respectively. “Norm" indicates whether the features were normalized before applying the mLR model, and “non-Norm" indicates that no normalization was applied. The corresponding cells in the table have not been filled since the liblinear solver is unable to handle the Multinomial scheme.

**Table 3:** Performance of logistic regression models—imbalanced dataset.

**Solver**	**Penalty**	**C**	**fit_intercept**	**Accuracy (SD) [%]**			
				**OvR**		**Multinomial**	
				**Non-Norm**	**Norm**	**Non-Norm**	**Norm**
lbfgs	None	–	F	Not conv	Not conv	61.18 (3.42)	Not conv
			T	Not conv	Not conv	66.45 (3.42)	Not conv
	L2	0.01	F	63.82 (2.87)	52.63 (4.16)	62.5 (3.89)	52.63 (4.16)
			T	63.82 (2.87)	52.63 (4.16)	61.84 (4.36)	52.63 (4.16)
liblinear	L1	0.01	F	70.39 (3.89)	27.63 (5.42)		
			T	70.39 (3.89)	27.63 (5.42)		
	L2	0.01	F	63.82 (2.87)	52.63 (4.16)		
			T	63.82 (2.87)	52.63 (4.16)		
Newton-cg	None	–	F	55.92 (10.59)	45.39 (1.14)	60.53 (4.92)	36.84 (2.63)
			T	55.92 (10.59)	40.79 (2.28)	59.87 (5.05)	42.76 (6.8)
	L2	0.01	F	63.82 (2.87)	52.63 (4.16)	62.5 (3.89)	52.63 (4.16)
			T	63.16 (1.86)	52.63 (4.16)	63.82 (3.89)	52.63 (4.16)
sag	None	–	F	63.82 (2.18)	40.13 (5.9)	63.82 (1.14)	38.16 (6.03)
			T	63.82 (2.18)	42.11 (5.58)	63.82 (1.14)	40.79 (5.43)
	L2	0.01	F	63.82 (2.18)	52.63 (4.16)	63.82 (1.14)	52.63 (4.16)
			T	63.82 (2.18)	52.63 (4.16)	63.82 (1.14)	52.63 (4.16)
saga	None	–	F	63.16 (4.16)	44.08 (6.8)	62.5 (2.18)	41.45 (7.06)
			T	63.16 (4.16)	43.42 (9.39)	62.5 (2.18)	44.08 (7.76)
	L1	0.01	F	62.5 (3.89)	27,63 (5.42)	61.18 (1.14)	27.63 (5.42)
			T	62.5 (3.89)	52.63 (4.16)	61.18 (1.14)	52.63 (4.16)
	L2	0.01	F	63.16 (4.16)	52.63 (4.16)	62.5 (2.18)	52.63 (4.16)
			T	63.16 (4.16)	52.63 (4.16)	62.5 (2.18)	52.63 (4.16)
	elasticnet	0.01	F	63.16 (4.92)	27.63 (5.42)	61.18 (1.14)	27.63 (5.42)
			T	63.16 (4.92)	52.63 (4.16)	61.18 (1.14)	52.63 (4.16)

The cells indicate the model's accuracy, accompanied by the standard deviation in parentheses, for each configuration under both OvR and multinomial strategies, with or without normalization. In some cases, results indicate “Not conv," meaning the model did not converge for that specific configuration. The use of different solvers and penalties affects convergence and accuracy, suggesting that specific parameter choices can enhance the model's classification performance.

Experiments with normalized data yielded weaker results. However, with non-normalized data, the modal value of accuracy of 63.82% is comparable to that of physician experts, slightly lower than that of advanced experts (Accuracy = 65%), and predominant over that of beginner physicians, who achieved an average accuracy of 58.42%. These values were calculated based on the study by Mesejo et al. (12).

In the “saga" solver case, the results obtained using the “L2" penalty are slightly ahead of those obtained using the “L1" penalty, but they remain equivalent to the scenario where no regularization was applied. We can conclude that for this solver, regularization is not necessary. Similarly, the “sag" solver does not justify the computing cost needed for regularization. The application of “L2" regularization in the case of the “newton-cg" solver leads to a slight increase in accuracy, which justifies its use. In contrast, the “lbfgs" solver in the “OvR" scheme fails to converge without regularization. In the “Multinomial" scheme with the “lbfgs" solver, the absence of regularization, combined with demanding a bias term, resulted in the second-best model in terms of accuracy.

The best results were obtained using the “liblinear" solver with the ‘L1" regulariser, achieving an accuracy of 70.39%; this solver remained unaffected by the presence of a bias term with the existing data. For non-normalized data, the lowest accuracy, of 55.92%, was observed for the “newton-cg" solver, without regularization and bias term.

The relatively small standard deviation values reflect the fact that the data in the dataset were well-shuffled.

In the Table 4, the results of the 88 models tested on the balanced dataset are presented. The meaning of the table heading is the same as the Table 3.

**Table 4:** Performance of logistic regression models—imbalanced dataset.

**Solver**	**Penalty**	**C**	**fit_intercept**	**Accuracy (SD) [%]**			
				**OvR**		**Multinomial**	
				**Non-Norm**	**Norm**	**Non-Norm**	**Norm**
lbfgs	None	–	F	Not conv	41.45 (4.31)	61.84 (3.95)	36.84 (6.17)
			T	Not conv	41.45 (4.31)	59.87 (5.05)	38.16 (3.95)
	L2	0.01	F	Not conv	48.03 (7.76)	Not conv	46.71 (9.74)
			T	Not conv	44.08 (10.09)	Not conv	44.74 (10.69)
liblinear	L1	0.01	F	64.47 (7.78)	27.63 (5.43)		
			T	64.47 (7.78)	27.63 (5.43)		
	L2	0.01	F	61.18 (2.87)	52.63 (4.16)		
			T	61.84 (2.28)	52.63 (4.16)		
Newton-cg	None	–	F	55.92 (10.59)	38.82 (5.99)	59.87 (5.05)	41.45 (7.30)
			T	55.92 (10.59)	39.47 (4.16)	59.87 (5.05)	41.45 (6.55)
	L2	0.01	F	60.53 (0.00)	48.68 (8.43)	62.5 (4.70)	46.71 (9.74)
			T	57.89 (3.22)	44.08 (8.60)	Not conv	43.42 (9.40)
sag	None	–	F	52.63 (3.72)	34.21 (4.92)	52.63 (3.22)	39.47 (6.17)
			T	52.63 (3.72)	34.87 (6.55)	52.63 (3.22)	37.50 (4.70)
	L2	0.01	F	52.63 (3.72)	48.03 (7.76)	52.63 (3.22)	46.71 (9.74)
			T	52.63 (3.72)	44.74 (9.30)	52.63 (3.22)	43.42 (9.40)
saga	None	–	F	52.63 (3.72)	33.55 (7.06)	51.32 (4.74)	34.22 (3.72)
			T	52.63 (3.72)	35.53 (4.74)	51.32 (4.74)	33.55 (2.18)
	L1	0.01	F	51.32 (4.74)	27,63 (5.43)	50.00 (5.58)	27.63 (5.43)
			T	51.32 (4.74)	36.84 (1.50)	50.00 (5.58)	44.74 (1.70)
	L2	0.01	F	52.63 (3.22)	48.03 (7.76)	51.32 (4.74)	46.71 (9.74)
			T	52.63 (3.22)	44.74 (9.30)	51.32 (4.74)	43.42 (9.40)
	elasticnet	0.01	F	51.32 (4.74)	27.63 (5.43)	51.32 (4.74)	27.63 (5.43)
			T	51.32 (4.74)	46.12 (1.35)	51.32 (4.74)	44.08 (1.21)

As can be seen in Table 4, the accuracy values decreased for all models, as expected. The accuracy value (70.39%) for the imbalanced dataset decreased to 64.47% for the balanced dataset. In the following, the accuracy is comparable to those of physician experts. Also, for the normalized values of the dataset, the accuracy obtained is lower than for the non-normalized values.

The previous conclusions (imbalanced dataset) regarding the use of regularizers remain valid also in the case of the balanced dataset.

### 3.2 Comparison with other methods and physician experts

In the subsequent discussion, we aimed to compare our two optimized models with the best performing model of Mesejo et al. (12), called Random Subspace and physicians.

The paper presents gastroenterologists opinion, with two knowledge levels: three beginner and four expert physicians, as well as the ground truth (i.e., histological classification).

When physicians perform a colonoscopy, they possess additional information, such as the location (whether it's in the ascending or descending colon), the orientation (whether they are facing forward or backward), along with the device, besides the photos, they can also maneuver to observe from different angles.

The distinction between expert and beginner gastroenterologists was based on the number of correct annotations provided during the evaluation phase. Specifically, we used the accuracy of polyp classification against the ground truth to classified instances. The ground truth was established by physicians, who likely had access to the data collected over approximately three years. This allowed them to observe the disease's progression over time, thereby establishing the truth with nearly 100% accuracy. Yet, when only presented with photographs, in the same manner as ML algorithms, these are the accuracies they achieved, based solely on the pictures without any additional information. Those whose performance exceeded 65% accuracy were designated as experts, while those below this threshold were considered beginners. This criterion was chosen post hoc to reflect observed diagnostic consistency and was applied uniformly across all physicians evaluators.

**Figure 3:**
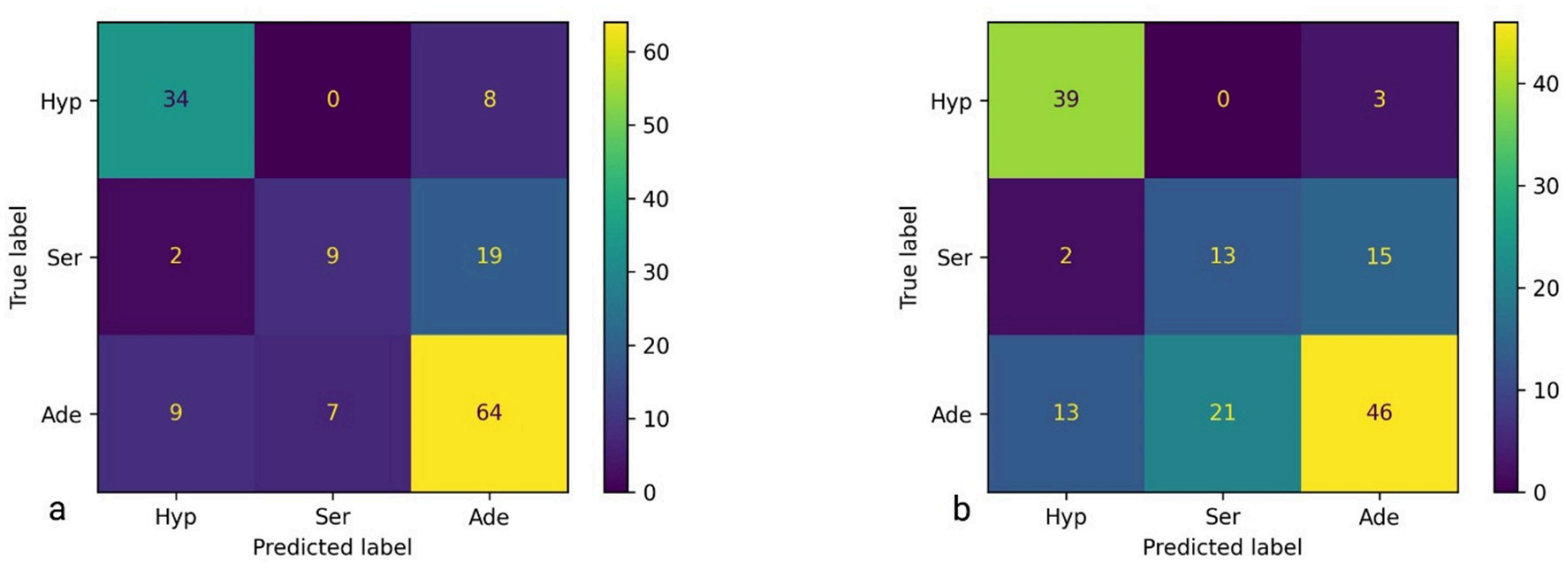
Confusion matrices for the best model (liblinear, L1 penalty, *C* = 0.01)—**(a)** imbalanced dataset—left side; **(b)** balanced dataset—right side.

The confusion matrices, presented in Figure 3, reflect the performance of the best classification model applied to the dataset, imbalanced and balanced, respectively. The matrices include predictions for the three classes: Hyperplastic (Hyp), Serrated (Ser), and Adenoma (Ade).

Matrix interpretation. The confusion matrix for the imbalanced dataset is structured with the following values:

- (Hyp, Hyp): 34 — The model correctly predicted 34 instances as belonging to the *Hyp* class.
- (Hyp, Ser): 0 — The model incorrectly predicted 0 instances of the *Hyp* class as *Ser*.
- (Hyp, Ade): 8 — The model incorrectly predicted 8 instances of the *Hyp* class as *Ade*.
- (Ser, Hyp): 2 — The model incorrectly predicted 2 instances of the *Ser* class as *Hyp*.
- (Ser, Ser): 9 — The model correctly predicted 9 instances as belonging to the *Ser* class.
- (Ser, Ade): 19 — The model incorrectly predicted 19 instances of the *Ser* class as *Ade*.
- (Ade, Hyp): 9 — The model incorrectly predicted 9 instances of the *Ade* class as *Hyp*.
- (Ade, Ser): 7 — The model incorrectly predicted 7 instances of the *Ade* class as *Ser*.
- (Ade, Ade): 64 — The model correctly predicted 64 instances as belonging to the *Ade* class.

The interpretations confusion matrix for the balanced dataset is similar to that explained above for the imbalanced dataset.

For imbalanced case the model achieved a performance score of 70.39, indicating a reasonable level of accuracy, with a standard deviation of 3.89. However, there are notable misclassifications, particularly between the *Ser* and *Ade* classes. The highest values appear on the main diagonal, reflecting a higher number of correct predictions. Nonetheless, the presence of significant values of the diagonal suggests that the model has difficulty distinguishing between certain classes, especially between *Ser* and *Ade*.

Can be observed in the confusion matrix of the balanced dataset that, although the accuracy, as a global metric, has decreased (from 70.39 to 64.47%), in fact, the separation power of the serrated and hyperplastic classes has increased. The optimized algorithm managed to correctly predict 8.6% serrated images in relation to 5.9% images as predicted by the same model for the imbalanced dataset. In addition, correctly predicted 4% more hyperplastic images than in the imbalanced case. Indeed, on the other hand, the predictive power of the adenoma class decreased from 42 to 30%.

As it is known that hyperplastic polyps are non-dangerous, they do not require resection (12). Thus, neither the patient is subjected to an unpleasant medical maneuver, nor does the physician waste time with a useless intervention. Instead, both adenoma and serrated polyps should be resected. The adenoma because is already carcinogenic, and the serrated because it has a high probability of becoming cancerous. As seen in the confusion matrix of the imbalanced dataset, the best model guesses 34 hyperplastic polyps out of 42 (i.e. 80.95%). This translates into the fact that the other eight hyperplastic polyps, wrongly classified as adenoma, based on this algorithm, the physician will receive the suggestion to resect them, although there is no need. On the other hand, we see that nine adenoma and two serrated polyps, are wrongly classified as hyperplastic. This means that the physician will leave 11 dangerous polyps not resected. For the balanced dataset, the algorithm guesses 39 out of 42 hyperplastic polyps, suggesting that only three polyps are resected from mistake. Instead, it classifies 15 dangerous polyps as hyperplastic (four more than in the previous case), leaving them not resected. As it is better to resect healthy polyps than to leave carcinogenic or potentially carcinogenic polyps not resected, the conclusion is that mLR cannot be used as a support software in making resection/non-resection decisions, neither in the imbalanced dataset nor in the balanced dataset version.

This performance, alongside the confusion matrix, implies that while the model performs fairly well, further refinement or alternative approaches might be necessary to increase its accuracy, despite the use of hyperparameters such as *liblinear, L1, True*, and *False*.

Table 5 offers a comparative analysis of the performance metrics for the mLR model applied to the imbalanced dataset (*mLR*^*i*^) and to the balanced dataset (*mLR*^*b*^), such as accuracy, precision, recall, and F1 scores across other models and physician expertise levels. Comparing the confusion matrices of experts and beginners, given in Mesejo et al. (12), it is observed that the standard deviation of the experts is smaller than that of the beginners, which means a lower risk of misclassification. In the case of the adenoma class, the average True Positive of beginners [26.7 (8.6)] is higher than the average True Positive of experts [25.5 (2.4)]. The corresponding standard deviation is noted between the right brackets. For verification, if there is a statistically significant difference between the means, we have applied the two-tailed *T*-test with Welch correction (52, 53). Obtaining *p*-value = 0.836 (Welch's approximate *t* = 0.235) indicates that there is no statistical difference. When two datasets have the same mean but different standard deviations, the dataset with the lower standard deviation indicates lower risk. Compared to the best algorithms (Random Subspace and SVM) of Mesejo et al. (12), mLR shows a lower recall for the *Ser* class, but is competitive in other areas. Instead, compared to physician experts, mLR performs better overall, particularly in accuracy and recall for the *Ade* class. The mLR model provides a reasonable and competitive performance, especially in terms of accuracy and precision, though it struggles with recall for the *Ser* class. Its balanced performance across different metrics makes it a robust choice, particularly when compared to physician performance at the beginner level. However, there may still be room for improvement, particularly in recalling the *Ser* class.

**Table 5:** Comparative performance.

**Metric**	***mLR* ^*i*^**	***mLR* ^*b*^**	**Mesejo2016**	**Experts**	**Beginners**
Acc Mean	—	—		65.00%	58.42%
Acc Max	70.39%	64.47	76.69%	—	—
Prec Hyp	**75.55%**	72.22%	66.67%	62.00%	66.26%
Prec Ser	56.25%	*38.24*%	69.23%	52.20%	34.54%
Prec Ade	70.33%	71.88%	80.56%	73.50%	66.75%
Recall Hyp	80.95%	**92.86**%	85.71%	67.94%	52.38%
Recall Ser	*30.00*%	*43.33*%	60.00%	63.76%	44.67%
Recall Ade	**80.00%**	*57.50*%	72.50%	63.75%	66.75%
F1 Hyp	**78.16%**	**81.25**%	75.00%	64.84%	58.51%
F1 Ser	39.13%	40.63%	64.29%	57.40%	38.95%
F1 Ade	74.85%	*63.89*%	76.32%	68.27%	66.75%
macro_av Pre	67.38%	60.78%	72.15%	62.56%	55.85%
macro_av Recall	63.65%	64.56%	72.74%	65.15%	54.60%
macro_av F1	64.05%	61.92%	71.86%	63.51%	54.74%

*mLR*^*i*^—our model applied to the imbalanced dataset; *mLR*^*b*^—our model applied to the balanced dataset. Bold indicates the best and *italic* the worst results.

Comparing the results obtained by applying LR on the imbalanced (*mLR*^*i*^) and balanced (*mLR*^*b*^) datasets, respectively, we notice that the accuracy increases very slightly only in the case of Ade polyps (from 70.33 to 71.88%), and Recall increases for Hyp and Ser ones (from 80.95 to 92.86% and from 30 to 43.33%, respectively). F1-score, as a harmonic mean of Precision and Recall, shows us a global increase of the two metrics. Even if the accuracy decreases in the case of balanced data compared to imbalanced data, overall, F1-score increases both for hyperplastic and for serrated. For the Hyp polyps, F1-score increases from 78.16 to 81.25%, and for the Ser ones, from 39.13 to 40.63%. Macro-Average shows that the only metric that increased in arithmetic average in the case of balanced data compared to imbalanced data is the Recall. However, the increase is small, from 63.65 to 64.56%.

### 3.3 Evaluation of additional classifiers and comparative evaluation with logistic regression

Following the analysis of mLR, we extended the classification framework to include four additional ML algorithms: RF, KNN, SVM, and XGBoost. These models were selected based on their demonstrated performance in biomedical classification tasks for structured data obtained from medical imaging.

All classifiers were trained on a feature matrix comprising 698 descriptors extracted from colorectal polyp images. The models were not applied directly to raw image data; instead, they operated on pre-extracted features, ensuring interpretability and computational efficiency. CNNs and other deep learning models are frequently used for image-level classification, but they were purposefully left out of this work in order to keep the focus on interpretable methods appropriate for medical applications with limited resources. However, CNNs may be taken into consideration in further research as a preliminary to automated feature extraction.

For each classifier parameter tuning was applied using grid search and performed stratified cross-validation, for instance, Random Forest was optimized with n_estimators=100 and max_depth=5, while XGBoost used n_estimators=100, max_depth=5, and learning_rate=0.1. SVM employed an RBF kernel with C=1.0 and gamma='scale', and kNN was configured with n_neighbors=5.

Lesion classification was approached using both multiclass and binary tasks. In the multiclass setting (Hyperplastic, Serrated, Adenoma), XGBoost achieved the highest performance, with a macro-average F1-score of 0.88, outperforming Logistic Regression (0.74), Random Forest (0.85), SVM (0.83), and kNN (0.56). The confusion matrix revealed high recall for adenomas (0.86) and a clear separation between lesion types.

**Figure 4:**
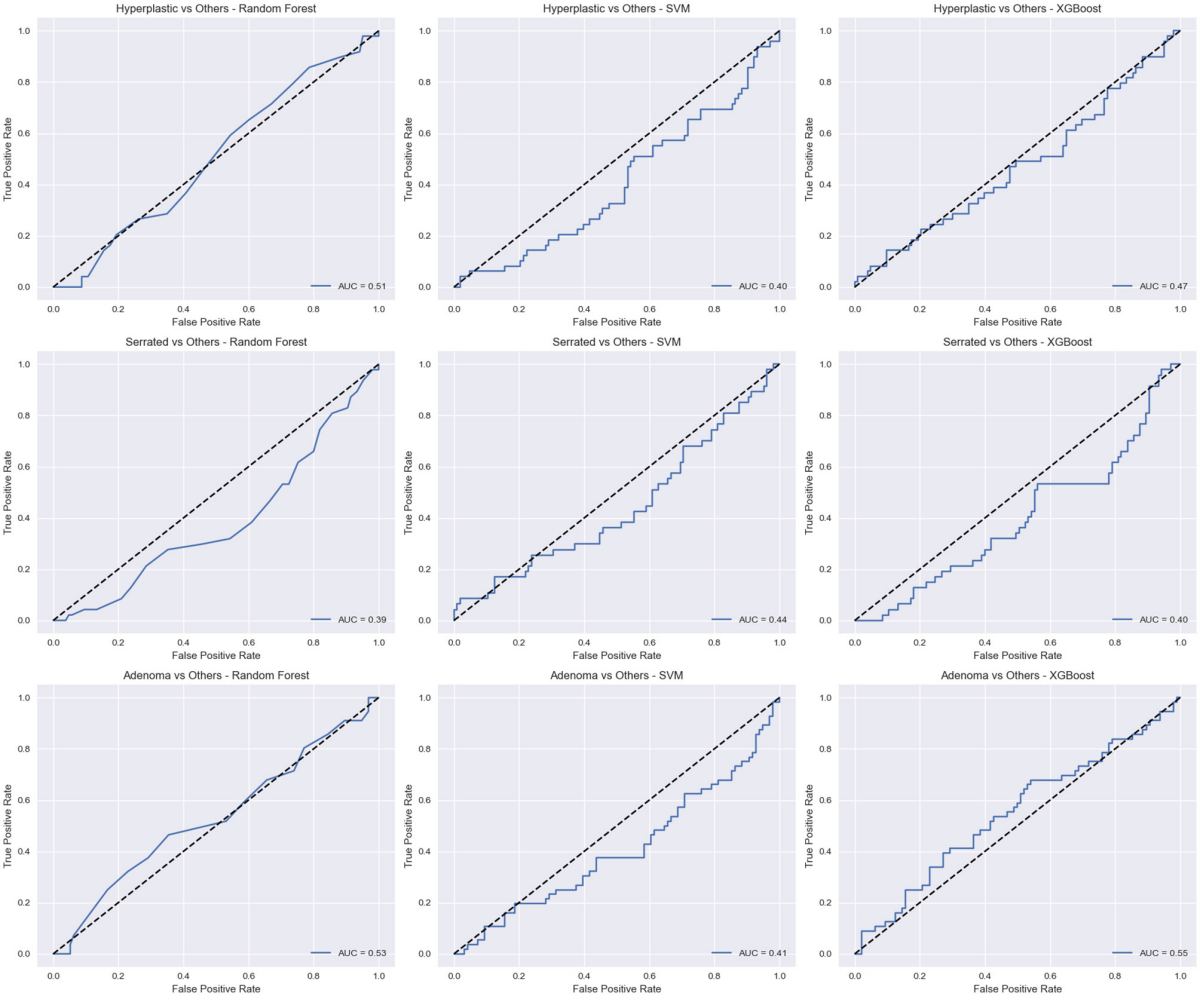
Classification report and ROC AUC curves.

For clinical validation, three binary classification tasks were formulated: Hyperplastic vs Others, Serrated vs Others, and Adenoma vs Others. ROC curves (Figure 4) demonstrated that XGBoost achieved AUC ≥ 0.90 across all three tasks, with peak performance for Adenoma (AUC = 0.94, F1 = 0.90). SVM and Random Forest also produced competitive results, though marginally lower.

Table 6 tive summary of classifier performance alongside physician benchmarks. XGBoost outperformed both mLRi/mLRb and the diagnostic accuracy of experts (65%) and beginners (58.42%) for automated lesion classification.

**Table 6:** Comparative performance across classifiers.

**Metric**	**Random Forest**	**SVM**	**kNN**	**XGBoost**
Acc Mean	**86.05%**	**84.21%**	**66.38%**	**89.34%**
Acc Hyp	89.47%	88.16%	69.08%	92.11%
Prec Hyp	88.89%	87.50%	60%	91.67%
Prec Ser	80%	88%	45%	88%
Recall Hyp	90%	88%	69.23%	93%
Recall Ser	84.21%	88.89%	17.39%	86.96%
Recall Ade	88.00%	86.00%	80.00%	90.00%
F1 Hyp	89.44%	87.58%	64.62%	92.00%
F1 Ser	81.00%	87.00%	24.00%	84.00%
Macro_av Pre	85.96%	87.83%	61.67%	90.56%
Macro_av Recall	87.40%	87.63%	56.87%	89.32 %
Macro_av F1	85.81%	87.19%	56.21%	88.67%

Bold values represent the overall accuracy (Acc Mean) for each classifier, used as the primary metric for comparative evaluation.

### 3.4 ROC AUC analysis

**Figure 5:**
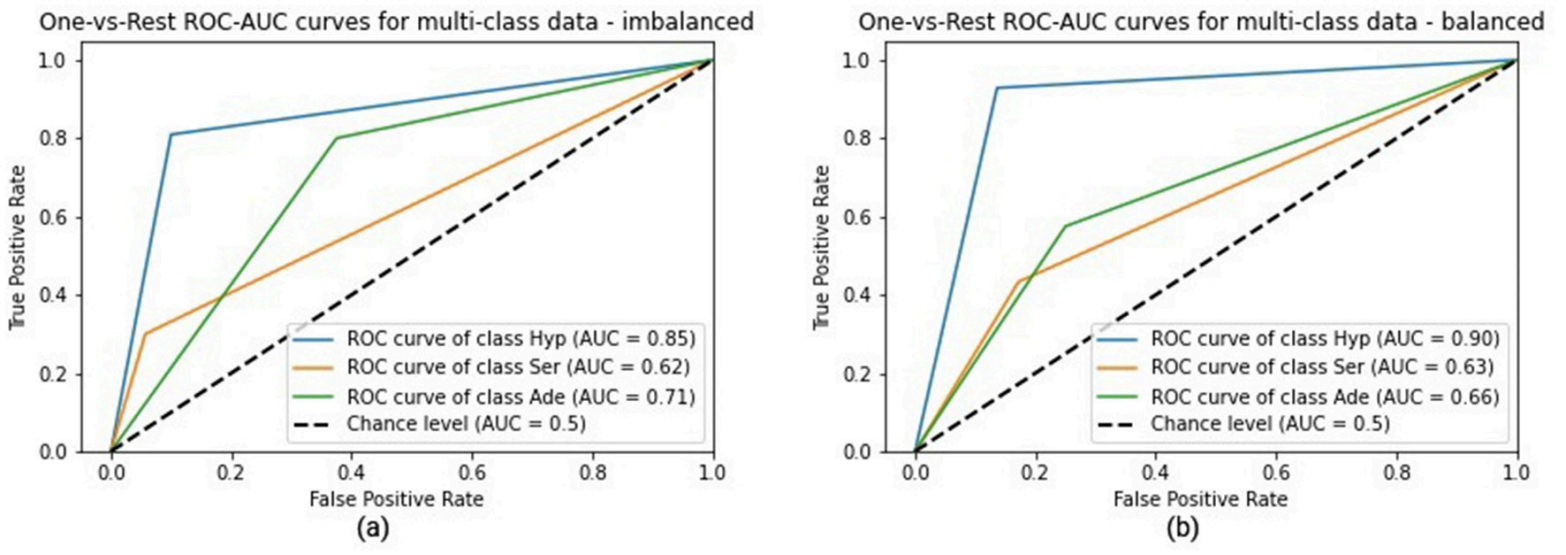
ROC AUC curves for the three classes; **(a)** imbalanced dataset—left side; **(b)** balanced dataset—right side.

In this work, we choose to use the OvR ROC AUC approach. The Figure 5a shows the ROC curves and AUC for the three classes (Hyp, Ser and Ade) being considered the best model applied to the imbalanced dataset, and the right side Figure 5b, for the balanced dataset. It can be seen that the AUC of the Hyp class increased from 0.85 (imbalanced data) to 0.90 (balanced data), the AUC of the Serr class remained approximately the same, and the AUC of the Ade class decreased from 0.71 to 0.66.

It is important to separate serrated polyps from the others, but the low AUC values (the lowest) show that the classification of serrated polyps is very difficult to achieve. The ROC curves of the hyperplastic class, as well as the high values of AUC (the highest for both variants: imbalanced and balanced dataset) show us that the algorithm can best detect these polyps compared to the others. We notice that the TPR has increased from 0.8095 (of the imbalanced dataset) to 0.9286 (for the balanced dataset), but at the same time, unfortunately, the FPR is also increasing from 0.10 to 0.136. In this classification, the cost of FPR is higher than the cost of TPR, which means that we prefer a decrease in TPR rather than an increase in FPR. The higher the TPR, the fewer healthy polyps are resected, and the higher the FPR, the more carcinogenic or potentially carcinogenic polyps are classified as hyperplastic. The decrease in TPR would not have been as faulty as the increase in FPR.

### 3.5 Statistical analysis of the best models

**Figure 6:**
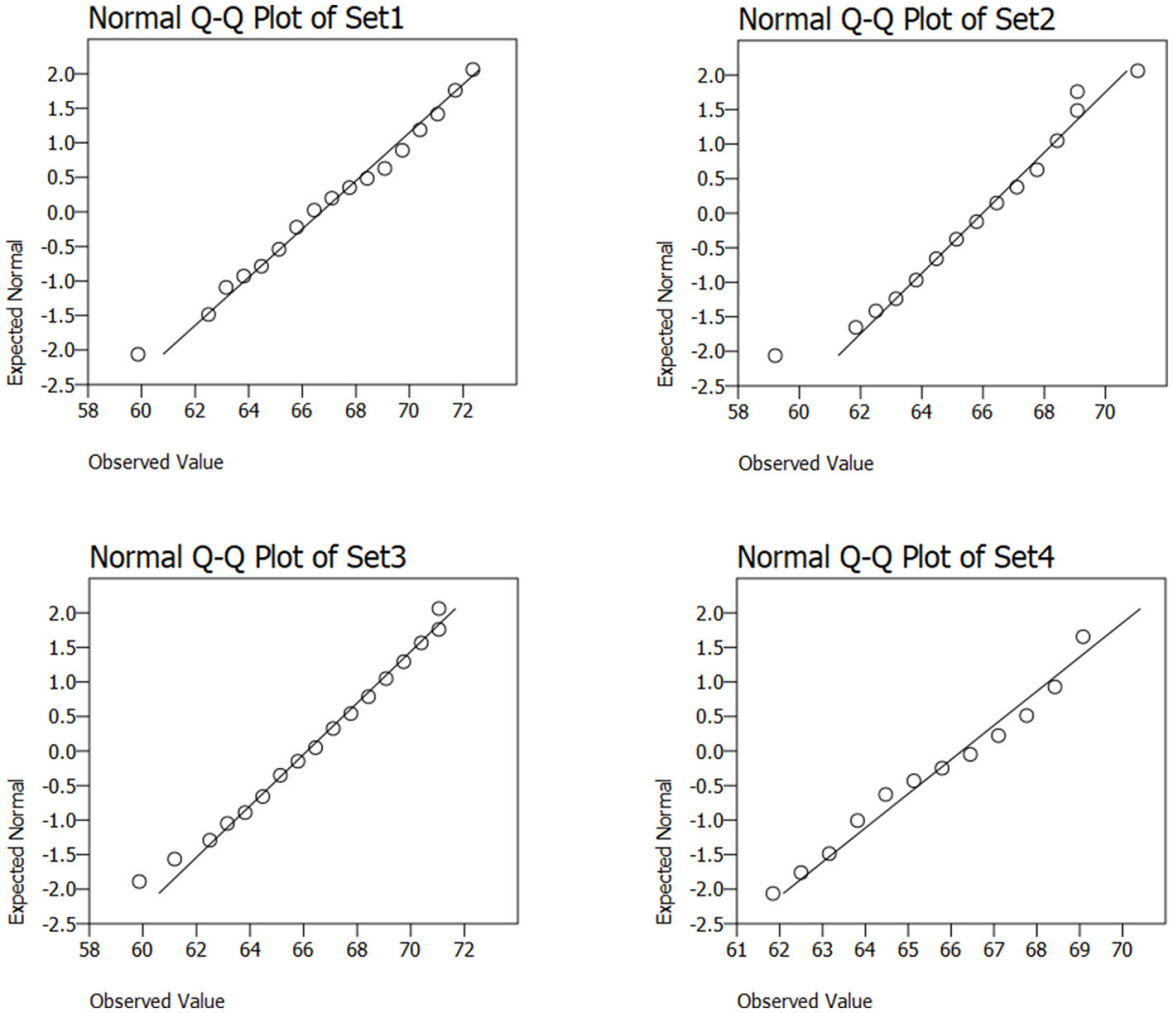
Q–Q Plots for visual validation of normality.

To assess the normality of the feature distributions, four representative subsets were selected from the extracted feature matrix and analyzed using Quantile–Quantile (Q–Q) plots in the Figure 6. These subsets, referred to as Set1, Set2, Set3, and Set4, correspond to distinct groups of features derived from different descriptor families:

- Set1: Texture-based descriptors (e.g., LBP, Color GLCM).
- Set2: Color-based descriptors (e.g., Color Naming, Opponent Color, Hue).
- Set3: Shape-based descriptors (e.g., S3D Shape, Kernel-PCA).
- Set4: Mixed descriptors with high variance and non-Gaussian behavior.

The Figure 6 revealed that Sets 1–3 shown approximate normality, with data points closely aligned along the diagonal reference line. In contrast, Set4 showed deviations at the distribution tails, suggesting possible deviation. The selection of classifiers and preprocessing techniques was influenced by these observations.

To check the influence of the data mix on the results obtained, for the best model (mLR with *liblinear* solver, *L1* penalty and *C* = 0.01), we performed 2 × 50 runs for *Fit_Intercept* = False and *Fit_Intercept* = True, respectively, where we mixed randomly the rows of the dataset at every run.

An unpaired two-tailed *T*-test was applied, considering the significance level α = 0.05. We suggest presenting this as a decision rule, as cited in Iantovics et al. (54) and Iantovics (55). The *p*-value = 0.166, with *p*>α, indicates no reason for rejecting the null hypothesis, indicating no statistical difference. The unpaired two-tailed *T*-test assumptions passed since both variables passed the normality assumption, verified using the Lilliefors test (*p*-value = 0.2, 0.2>0.05) and the two sample variances were equally verified using the *F*-test (*p* = 0.12, 0.12>0.05).

The results are presented following this methodology. If the decision rule cannot be applied due to assumptions failing, an alternative methodology may be considered (54). Generalizations for comparing more than two models are discussed in Iantovics et al. (56) and Iantovics (57).

The Graphical Abstract summarizes the workflow developed to classify polyps into three lesion types: hyperplastic, serrated, and adenoma. It is organized into three main sections *Input, Methods*, and *Output* and integrates both data flow and comparative performance metrics. The input displays the distribution of annotated polyps: 21 hyperplastic, 15 serrated, and 40 adenoma lesions. A total of 698 handcrafted features were extracted using eight descriptor families: AHT, LBP, Color Naming, Discriminative Color, Opponent Channel, Shape-DNA, and Kernel-PCA.The methods highlights the five ML classifiers evaluated: mLR, kNN, SVM, RF and XGBoost. A confusion matrix illustrates the performance of mLR across the three classes, showing correct and misclassified instances. A table summarizes the logistic regression configurations tested, including solvers, penalties, and regularization parameters. The output presents classification accuracies for each lesion type and model. XGBoost is visually emphasized as the best-performing model, achieving a macro-average F1-score of 0.88 and overall accuracy of 89%. It is followed by Random Forest (F1 = 0.85, accuracy = 86%), SVM (F1 = 0.83, accuracy = 84%), and kNN (F1 = 0.56, accuracy = 66%). These results are contextualized against physician benchmarks: experts (65%) and beginners (58.42%).

The figure encapsulates the core findings of the study, demonstrating the effectiveness of ensemble methods in automated lesion classification.

## 4 Discussions and limitations

This research demonstrates the potential of ML classifiers in polyp characterization using colonoscopy-derived features. While Logistic Regression offers clinical interpretability, ensemble methods such as Random Forest and XGBoost achieved superior performance, particularly in distinguishing adenomatous lesions. The inclusion of binary classification tasks and ROC analysis further strengthens the clinical relevance of the findings.

However, some limitations must be mentioned, firstly, the dataset is relatively small, which may affect generalisability. The selected dataset, comprising 152 instances corresponding to 76 annotated colorectal polyps, was chosen to represent a small-data diagnostic case, also because it is difficult to get big annotated datasets, particularly in initial implementations or uncommon lesion types. This constraint motivated the use of interpretable ML models capable of generalizing from limited samples. External validation on independent or cross-institutional datasets is still required, even if stratified five-fold cross-validation was used to reduce overfitting. Secondly, the study does not include deep learning models such as CNNs, which are typically applied directly to image-level data. Nevertheless, CNNs could be explored in future work as a preliminary step for automated feature extraction.

Thirdly, the claim that Logistic Regression outperforms physician experts requires cautious interpretation. While the model achieved higher average accuracy than beginners and experts, its misclassification of clinically significant polyps (e.g., adenoma as hyperplastic) limits its utility in resection decision support. Despite limitations in handling serrated polyps, the model provides valuable insights for real-time clinical applications.

Finally, the distinction between expert and beginner evaluators was based on clinical experience and certification, as detailed in Section 3.2. While performance differences were modest, this stratification provides a meaningful benchmark for automated systems.

## 5 Conclusions

Logistic Regression (LR) is easy to use and interpretable, making it a useful tool for initial exploratory analysis and for providing baseline performance metrics. Its efficiency in handling binary outcomes enables it to model the probability of polyp presence or absence based on extracted features from endoscopic images. However, the effectiveness of LR can be constrained by its inability to capture complex, non-linear relationships in high-dimensional medical image data.

In this study, we implemented and assessed the performance of mLR algorithms for classifying GI lesions, specifically utilizing the One-vs-Rest (OvR) and Multinomial LR classifiers for colon polyp image classification. The results obtained are comparable to the best results obtained by Mesejo et al. (RS) (12) and slightly surpass those obtained by physicians. Our findings demonstrate that while mLR provides a useful starting point for such classification tasks, further exploration is needed to improve model accuracy and robustness.

Future research will focus on refining the methodology to better accommodate the complexities inherent in medical image data. This will include exploring advanced algorithms capable of capturing non-linear relationships and integrating additional features that may enhance predictive performance.

Moreover, it is essential to summarize the methodologies employed in this study, particularly those based on the works of Iantovics et al. (48, 54–57), as these approaches have often been overlooked or misrepresented in existing literature. Addressing this aspect can lead to more robust research practices and prevent the publication of misleading findings, even in reputable journals. By emphasizing methodological clarity, we can contribute to more reliable outcomes in future studies involving medical imaging.

Future work will focus on improving the classification of serrated polyps, which proved challenging in the current study. We plan to explore the frequently used machine and deep learning algorithms, such as ensemble methods and CNN, which are well-suited for capturing the complex patterns in medical images. Additionally, more sophisticated feature extraction techniques, such as texture analysis or wavelet transforms, will be investigated to improve model sensitivity and accuracy for serrated polyps.

## Data Availability

Publicly available datasets were analyzed in this study. This data can be found in the paper by Mesejo P, Pizarro D, Abergel A, Rouquette O, Beorchia S, Poincloux L, et al. Computeraided classification of gastrointestinal lesions in regular colonoscopy. *IEEE Trans Med Imaging*. (2016) 35:2051–63.
